# Antenatal care utilization and compliance with national and WHO guidelines in rural Ethiopia: a cohort study

**DOI:** 10.1186/s12884-022-05171-3

**Published:** 2022-11-17

**Authors:** Meselech Roro, Wakgari Deressa, Bernt Lindtjørn

**Affiliations:** 1 Centre for International Health, University of Bergen, Bergen, Norway; 2 Department of Reproductive Health and Health Service Management, School of Public Health, College of Health Sciences, Addis Ababa University, Addis Ababa, Ethiopia; 3 Department of Preventive Medicine, School of Public Health, College of Health Sciences, Addis Ababa University, Addis Ababa, Ethiopia

**Keywords:** Antenatal care, Pregnancy outcome, Adverse pregnancy outcome, Compliance, Ethiopia

## Abstract

**Background:**

Antenatal health care utilization has the potential to influence maternal and new-born health. In this study, we assessed compliance of antenatal care utilization with national and World Health Organization (WHO) guidelines. We also examined association of antenatal care utilization with adverse pregnancy outcomes as secondary outcome.

**Methods:**

This was a community-based cross sectional study conducted from July 2016 to November 2017 in rural south-central Ethiopia. We described antenatal care received by pregnant women, whom we followed at three prescheduled visits during pregnancy and collected birth data at time of delivery. Extent of antenatal care content received, timing of antenatal care, place of antenatal care and place and mode of delivery were obtained and computed in accordance with national and WHO guidelines. For adverse pregnancy outcomes, computed as sum of low birth weight, preterm birth, intrauterine foetal death, and stillbirth, the exposure variable used was antenatal care utilization.

**Results:**

Seven hundred and four (704) women participated in the study, and 536 (76.1%) had attended at least one antenatal care visit. Among women who attended antenatal care visit, majority, 421 (79.3%), had done so at health centres and hospitals, while 110 (20.7%) attended at health post. Average number of antenatal care visits was 2.5, which is less than that recommended in national and WHO guidelines. Only 18 (2.6%) women had attended antenatal care in their first trimester, which is low in contrast to the expected 100% specified in the guidelines. Less than half (47%) of the women delivered in a health facility. This is in contrast to the 100% expected health institution deliveries. Low birth weight was 7.9% (*n* = 48), and preterm birth was 4.9% (*n* = 31). There were 12 twin pregnancies, three stillbirths, 11 spontaneous abortions, and two intrauterine foetal deaths. We did not find significant association between adverse pregnancy outcomes and antenatal care utilization (COR = 1.07, 95% CI 0.62, 1.86).

**Conclusion:**

This study showed that antenatal care service utilization in the study area was markedly low compared to that recommended in national and WHO guidelines. The obtained antenatal health care utilization was not associated with the registered adverse pregnancy outcomes.

**Supplementary Information:**

The online version contains supplementary material available at 10.1186/s12884-022-05171-3.

## Background

The benefit of antenatal health service utilization is the provision of evidence-based clinical interventions. These include maternal health education and counselling, examinations, and arranging specialized care when the need arises [1]. Antenatal care (ANC) provides an opportunity for influencing the future health and well-being of pregnant women, foetuses, and neonates. It assists to identify high-risk women to facilitate timely management during pregnancy and child birth [2].

According to the 2016 Ethiopian Demographic and Health Survey, maternal mortality ratio for Ethiopia was 412 maternal deaths per 100,000 live births. The proportion of women who received ANC from a skilled provider was 62% in 2016. Thirty-two percent of women had at least four ANC visits during their last pregnancy. Institutional deliveries was 26% in 2016. Thirteen percent of the new-borns had low birth weight [3].

Ethiopia has emphasised strengthening the provision of ANC services at all levels in the health system to facilitate the improvement of positive pregnancy outcomes [4]. The country’s health service is currently structured into a three-tier system: primary, secondary, and tertiary levels of care. Primary hospitals, health centres, and health posts are included under the primary level of care, while the secondary level comprises general hospitals serving 1 to 1.5 million people. Tertiary level health care is composed of specialized hospitals that provide services for 3.5 to 5 million people [5].

Ethiopia is implementing the World Health Organization (WHO) focused ANC (FANC) model at all health facilities. FANC is a goal-orientated approach to delivering ANC service four times during pregnancy. Women with low risk pregnancies need to have ANC visits at gestational ages between 8 and 12 weeks, between 24 and 26 weeks, at 32 weeks, and between 36 and 38 weeks. Women with high-risk pregnancies and those who develop pregnancy complications are required to have a greater number of ANC visits [4, 6].

According to the guidelines for ANC in Ethiopia, every pregnant woman is expected to receive ANC from a skilled provider. This includes a comprehensive physical examination, blood tests for screening of anaemia and infection, a urine test, tetanus toxoid injections, iron and folate supplements, and medications for deworming [4]. Community-based HEWs in their respective kebeles deliver the ANC service at health posts, which is part of the first level of primary health care unit. However, HEWs do not provide the services of blood pressure measurement, blood tests for screening of anaemia or infection (except for malaria), blood glucose level tests, and urine tests at the health posts. The HEWs do, however, also identify pregnant women in their catchment areas and link them to health centres and hospitals.

Several studies have examined the usefulness of ANC on the uptake of safe delivery care, and the reduction of maternal and infant mortality [7, 8]. An intervention study from southern Ethiopia showed that ANC is important to reduce maternal complications, maternal death, and neonatal deaths [9]. In Ethiopia, a national key indicator survey from 2016 reports that the ANC service utilization was 62% [8]. Moreover, only 20% of women had their first ANC during the first trimester. The proportion of women who had four ANC visits during their pregnancy was only 32% [10].

Studies from several countries have found that poor ANC attendance is an important risk factor for adverse pregnancy outcomes [11, 12]. As the number of ANC visits decreases, there was an increase in the risk of preterm birth and stillbirth [13–16]. Women who had no ANC follow-up and those who had an ANC visit less than four times during pregnancy were also found to have a low birth weight (LBW) baby [14, 17, 18].

Although the above studies report potential benefits of ANC on pregnancy outcomes, they possess several limitations. They were most often cross-sectional or retrospective studies or reports not published in peer-review journals. Covariates, such as wealth status, that may affect the outcome were not included in the analysis of these studies. In addition, scientific arguments on the association between ANC and pregnancy outcomes have continued and are unresolved. For instance, a review article on randomized trials and meta-analysis did not find any relationship between ANC follow-up and preterm birth [19–22].

The objective of this study was to assess the compliance of ANC utilization with national and WHO guidelines in rural south-central Ethiopia. In addition, we assessed if adverse pregnancy outcomes, including intrauterine foetal death, stillbirth, low birth weight and preterm birth, were associated with higher use of antenatal services.

## Methods

### Study design and settings

This cross sectional study was conducted in a rural community of south-central Ethiopia from July 2016 to November 2017. The data were gathered as part of a cohort study to monitor intrauterine growth and the association between intrauterine growth and linear growth of children [23, 24]. We assessed the compliance of ANC utilization with national [4] and WHO [6] guidelines, and examined the association between the utilization of antenatal health care services and adverse pregnancy outcomes. The present study is a part of a larger project on intrauterine growth and child growth in Ethiopia. Details of the methods of this study have been described elsewhere [23, 24].

This study was carried out in the Adami Tullu district in the East Shewa Zone of the Oromia Regional State in Ethiopia. In 2015, the projected population of the district was 177,390 people [25]. The livelihood of the majority of the population depends on subsistence agriculture or cattle-rearing, while some depend on fishing at a nearby lake, Lake Zeway [25].

There were 104 health extension workers (HEWs) in 43 health posts in the district in 2016. Based on the national Health Extension Program (HEP) that was launched in 2007, each of the 48 *kebeles* (lowest administrative level) in the district is intended to have at least one health post staffed by two female HEWs reporting to the health centre. The HEWs deliver key promotion and preventive health services through static and outreach programs. One of the services is family health care, which includes the promotion and provision of family health services, ANC, delivery services, postnatal care, family planning, and immunizations [26]. There was one public hospital, one non-governmental organization hospital, and 12 public health centres in the district in 2016. The health centre is primarily staffed by public health officers, nurses, midwives, pharmacists, and laboratory technicians.

### Study population

We recruited pregnant women at less than 24 weeks of gestation from July 2016 to June 2017 from all pregnant women living in the study area. The study participants were followed during pregnancy until the time of delivery. The pregnant women were having routine ANC at the health facilities. During the scheduled visits, we measured blood pressure, height and weight of the pregnant women, performed haemoglobin tests, and followed intrauterine foetal growth using ultrasound at the study sites. This was a part of a project and was performed in addition to any examinations at the routine ANC.

### Sample size estimation

We used the sample size calculated for another study on intrauterine foetal growth. The details of the sampling assumptions are described in detail elsewhere. In brief, a total sample size of 716 was calculated to estimate birth weight as an outcome measure [23].

In addition, we also tried to check the adequacy of the sample size for this specific paper. We estimated about 587 cases were required to detect difference in proportion of low birth weight among women who were attending ANC (13.2%) and those were not attending ANC (23.5%) [27].

### Variables

#### Outcome variables

Antenatal care utilization was collected by interviewing each pregnant woman at each visit. For those who had attended antenatal care, they were asked to state the number of antenatal visits that they had attended. The pregnancy outcomes measured were low birth weight, preterm birth, intrauterine foetal death, and stillbirth.

Low birth weight was categorized as “yes” or “no”. “Yes” was defined as a birth weight < 2500 g, and “no” was defined as a birth weight > =2500 g, as they are defined by the WHO. Preterm birth was defined as delivery at a gestational age of less than 37 weeks [28]. Intrauterine foetal death was defined as a foetus with no signs of life in utero on ultrasound examination. Stillbirth was defined as a baby born with no signs of life, such as beating of the heart, pulsation of the umbilical cord, or definite movement of voluntary muscles [29]. Adverse pregnancy outcomes in the statistical analysis are a composite variable that summarizes low birth weight, preterm birth, intrauterine foetal death, and stillbirth. Adverse pregnancy outcome was categorized as “yes” or “no”; “yes” if there were any of the adverse pregnancy outcomes, and “no” if none of them were present.

#### Exposure variables

Antenatal care utilization was one of the exposure variables. ANC use was categorized as “yes” or “no”; “yes” if the woman attended ANC, and “no” if she did not attend ANC.

Maternal characteristics (i.e., age, occupation, wealth status, height, education, gravida, parity, haemoglobin, and systolic and diastolic blood pressure) were also obtained.

### Data collection

Women in the reproductive age group were invited to participate in the study in selected kebeles by data collectors going house-to-house. Potential pregnancy was identified by using a pregnancy checklist [30] or as reported by the woman. Once pregnancy was suspected, the women were sent to a nearby health post or established study centre, where an ultrasound was performed to confirm the presence of pregnancy. All pregnant women with a gestational age of less than 24 weeks were invited to participate in the study. After the women had given written informed consent and were enrolled in the study, the pregnant women were given a card with an identification number and dates for three prescheduled consecutive visits at 26, 30, and 36 weeks of gestation at the data collection sites. More details are described in a previous publication [23].

Data at these three visits were mostly collected at health posts where routine ANC is provided. For those women who lived more than one kilometre away from the health post, a temporary station was established for the purpose of the study, in communication with the respective kebele managers. *Trained data collectors (nurses)* who were overseen by trained supervisors collected data on socio-demographic variables, past and present obstetric history, and health care service utilization. The supervisors, in addition, were communicating with respective authorities: woreda (district) health offices, kebele administrative offices, and HEWs at health posts. The HEWs were involved in facilitation of the data collection, as well. The pregnant women were advised to follow the routine ANC service provided during pregnancy at nearby health facilities. When any abnormal finding of the pregnant woman or the foetus was detected during data collection, they were referred to a nearby hospital.

A structured and pre-tested tool was used to collect data. The data were collected at enrolment and at each visit by 13 nurses whom we trained on data collection for the study*.* They conducted interviews, and took maternal and new-born anthropometric measurements. The nurses were given skill-based training on how to take anthropometric measurements prior to the data collection. The first author, who is a midwife and public health professional, was trained and performed the ultrasound examinations.

At enrolment, maternal anthropometric, obstetric, and sociodemographic data were collected. At the subsequent scheduled visits, we collected data on ANC utilization, pregnancy status, anthropometric measurements, blood pressure, ultrasound examinations, and haemoglobin measurements. At birth, we collected data on date of delivery, birth weight, and pregnancy outcome. We followed all of the study participants, except those loss to follow-up (3.2%), until delivery.

### Antenatal care service utilization

We interviewed each pregnant woman at each visit, irrespective of whether or not she had been attending antenatal care. Information on antenatal care (ANC) service utilization was collected at the three periods of follow up during pregnancy. The three periods were during 24 to 28 weeks of gestation, 29 to 33 weeks of gestation, and 34 to 38 weeks of gestation. At each visit, we asked if she had attended ANC, the place of the ANC attendance, the iron and folic acid supplementation (IFAS) received there, and duration of IFAS. Information on the status of pregnancy and birth outcome were collected during pregnancy and at delivery. The nurse data collectors visited those pregnant women who were expected to deliver at home after 36 weeks of gestation regularly until they gave birth. We then compared these ANC utilizations with the national and WHO guidelines.

### Measurement of pregnancy outcomes

Data on each outcome of pregnancy were collected by maternal interview, ultrasound assessment, and at delivery. The study was completed when the last enrolled pregnant woman delivered in November 2017. At birth, date of delivery and new-born birth weight were recorded by trained nurses within 72 hours after delivery, either at home or at a health care facility. Birth weights were measured using a digital infant weighing scale (Beurer Digital Baby Scale). Those pregnancies not ending in live birth and with no sign of life at birth were also recorded. Intrauterine deaths that occurred before birth were recorded during follow-up of the pregnancies.

We trained the data collectors (nurses) for 2 days, and the measurement techniques were standardized. To standardize the measurement techniques for maternal height, we checked the intra- and inter-tester technical errors of measurements (TEMs). We gave re-trainings until the measurement was within the recommended cut-off points [31]. For height, the intra-tester TEM was 0.38 cm, and the inter-tester TEM was 0.37 cm. We used a standard wooden board for the measurement of the height of each woman at enrolment.

### Ultrasound measurements

At enrolment, we calculated gestational age and estimated date of delivery based on ultrasound measurement of biometric parameters, using the Hadlock et al. formula [32]. To estimate gestational age and expected date of delivery, we used early sonographic estimation of dating, as it is reliable when the estimation is done before 24 weeks of pregnancy [33]. The ultrasound machine that we used throughout the study was the same. The first author, who was trained by a senior obstetrician, performed all of the ultrasound examinations. Quality and accuracy of the measurements were validated. The details are presented in a previous study [23].

### Statistical analysis

Data were entered and cleaned using SPSS version 24 (SPSS, Chicago, IL, U.S.A.). Then, they were exported to Stata software version 15 (Stata Corp., College Station, TX, U.S.A.) for further analysis. Descriptive statistics, frequency, percentage, mean, and standard deviation were computed.

In this study, the statistical methods used were for a cross-sectional study as we summarised the outcomes for the whole duration of the pregnancy. The outcome variables of low birth weight, gestational age at delivery, spontaneous abortion, intrauterine foetal deaths and stillbirth were assessed, and their proportion was calculated. The extent of ANC content received, timing of ANC, place of ANC, and place and mode of delivery were computed in accordance with the national and WHO guidelines. The frequency of ANC service utilization was the total visits each women had during the follow up.

Adverse pregnancy outcome, a composite variable, was computed as the sum of low birth weight, preterm birth, intrauterine foetal death, and stillbirth. Binary logistic regression analyses was used to identify the association of antenatal care service utilization on adverse pregnancy outcome. Crude odds ratio with 95% confidence interval (CI) was calculated. *P*-value *<* 0.05 was considered statistically significant.

## Results

### Description of the study population 

**Fig. 1:**
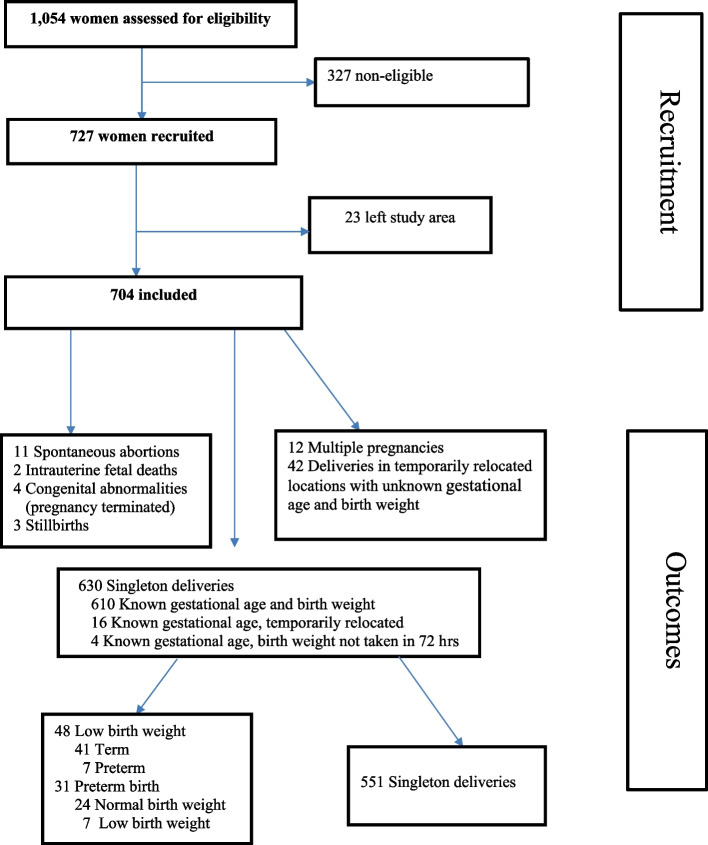
Flowchart of study participants included in the recruitment and outcomes of the study

There were 1054 self-reported pregnant women during the initial screening. From these, 727 met the criteria for inclusion in the study (Fig. 1). Eleven [11] of them were found to be not pregnant, and 316 of them were pregnant with more than 24 weeks of gestation, on ultrasound examination. Those women who left the study area, i.e., 23 (3.2%), before the follow-up period were excluded from the final analysis. We included 704 pregnant women who were enrolled prior to the 24th week of gestation in the final analysis (Fig. 1).

From the 704 pregnant women that we followed, 12 had a twin pregnancy, 11 had an abortion, two had an intrauterine foetal death, and four had congenital abnormalities. At delivery, three of the pregnancies ended in stillbirth. Birthweight was measured within 72 hours of delivery for 610 new-borns, and date of delivery was recorded for 630 new-borns. Four new-borns had a known date of delivery, but their birth weight was not measured within 72 hours of delivery. Sixteen [16] of the new-borns of pregnant women who were temporarily relocated for delivery to another place had a known date of delivery. Both date of delivery and birth weight were not known for 42 new-borns of the pregnant women who were temporarily relocated to another place for delivery. There were 48 low birth weight new-borns, among whom seven were preterm births. There were 31 preterm births, of whom 24 had a normal birth weight (Fig. 1).

### Characteristics of study participants

#### Maternal characteristics

The mean age of the women studied was 25 years. Out of the 704 study participants, 317 (45.0%) could not read or write, and only 60 (8.5%) of them attended secondary education. Their wealth status in the three categories was almost the same: poor (230 (33.0%)), middle (237 (34.0%)), and rich (231 (33.0%)).

The mean (Standard Deviation (SD)) maternal height was 157.3 (6.4) cm. We found anaemia in 135 (19.4%) of the women during pregnancy. One (0.4%) of them had severe anaemia, 31 (4.4%) had moderate anaemia, and 103 (14.6%) had mild anaemia. The majority of the women (540 (76.7%)) were housewives in occupation. One hundred and forty-one (141 women; 20.0%) of them were prim-para, and 431 (61.2%) were multipara (Table 1).

**Table 1:** Maternal characteristics of study participants (*n* = 704) in the Adami Tullu district, 2018

Variable	Frequency (%)	Mean (SD)
**Maternal characteristics**		
Maternal age (yr)		25 (5)
< 20	107 (15.2)	
20–24	238 (33.8)	
25–34	312 (44.3)	
35 and above	47 (6.7)	
Educational level		
Illiterate	317 (45.0)	
Read and write	43 (6.1)	
Primary	284 (40.3)	
Secondary	60 (8.5)	
Occupation (type)		
Housewife	540 (76.7)	
Farmer	120(17.0)	
House maid	17 (2.4)	
Others	27 (3.9)	
Wealth status (index)		
Poor	230 (33.0)	
Middle	237 (34.0)	
Rich	231 (33.0)	
Maternal height (cm)		157 (6)
< 150	73 (10.5)	
150–159	371(53.2)	
160 and above	253 (36.3)	
Gravidae (number)		
1	113 (16.1)	
2	142 (20.2)	
3	103(16.1)	
4	78 (11.1)	
5 and above	268 (38.0)	
Parity (number)		
0	132 (18.8)	
1	141 (20.0)	
2	101 (14.3)	
3	76 (10.8)	
4	79 (11.2)	
5 and above	175 (24.9)	
Systolic blood pressure (mmHg)		101.7 (10.1)
> = 140	1(0.4)	
120–139	28 (4.0)	
< 120	675 (95.6)	
Diastolic blood pressure (mmHg)		66.7 (8.1)
> = 90	0 (0)	
80–89	41 (5.8)	
< 80	663 (94.2)	
Anaemia (haemoglobin in g/dl)		11.9 (1.1)
Severe (< 7 g/dl)	1 (0.4)	
Moderate (7–9.9 g/dl)	31 (4.4)	
Mild (10–10.9 g/dl)	103 (14.6)	
None (> 11 g/dl)	569 (80.8)	

### Antenatal care utilization characteristics

Most of the women (536, 76.1%) had attended at least one ANC follow-up. From those who had been attending ANC, most (408, 77.1%) had two or more ANC follow-ups. Among the pregnant women who attended ANC, only 110 (20.7%) of them reported to have attended at a health post. From the study participants, 247 (35.1%) got IFAS during their pregnancy. The majority (143 57.9%)) of them took IFAS for 1 month. Twenty-four (24 (3.4%)) of the pregnant women reported having had a test for syphilis, and 202 (28.7%) of them reported having had a test for Human Immunodeficiency Virus (HIV). Three hundred and fifteen (315 (44.7%)) of the pregnant women reported having received a tetanus toxoid vaccination during the current pregnancy.

The proportion of women who attended antenatal care in all kebeles was similar, and there was no significant difference in attendance of antenatal care (X^2^ = 17.03, *P*-value = 0.149) among the kebeles (Supplementary Table 1). Moreover, antenatal care utilization showed no significant difference among sociodemographic and obstetric characteristics of women (Supplementary Table 2). When asked about the intended place of delivery, 480 (76.2%) of the women’s intention was a health care facility. However, only 293 (46.9%) of the women gave birth at a health care facility. A significant discrepancy was found between their intention and actual place of delivery (X^2^ = 22.58, *p* = 0.001). From those women who had an intention to deliver at a health facility, approximately 40% of them delivered at home. Only eight (1.3%) of the women delivered by caesarean section, while four (0.6%) had vacuum delivery. The mode of delivery in all of the rest (98.1%) was spontaneous vaginal delivery. During delivery, 235 (44.3%) of the women were assisted by traditional birth attendants (Table 2).

**Table 2:** Characteristics of antenatal care utilization (n = 704 pregnant women), Adami Tullu district, 2018

Variable	Frequency (%)
Antenatal care follow-up (at least one)	
Yes	536 (76.1)
No	168 (23.9)
Frequency of ANC (number)	
1	123 (22.9)
2	180 (33.6)
3	204 (38.1)
4	24 (4.5)
Place of ANC visit (type)	
Health post	110 (20.5)
Health centre and hospital	426 (79.5)
Iron and folic acid supplementation	
Yes	247 (35.1)
No	457(64.9)
Duration of iron and folic acid supplementation	
One month	143 (57.9)
Two or more months	104 (42.1)
Intended place of delivery	
Home	150 (23.8)
Health institution	480 (76.2)
Place of delivery (type)	
Home	333 (53.1)
Health institution	293 (46.9)
Mode of delivery (type)	
Spontaneous vaginal delivery	609 (98.1)
Assisted delivery	4 (0.6)
Caesarean Section	8 (1.3)
Assistant at birth (type)	
Traditional birth attendant	235 (44.3)
Health professional	269 (50.8)
Friends and/or relatives	19 (1.6)
The woman herself	7 (1.3)

### Pregnancy outcome characteristics

Six hundred and ten (610) new-borns were included for the birth weight estimation. The mean birth weight (SD) was 3214 (525) gm. Four hundred and thirteen (413 (67.7%)) had a birth weight of more than 3000 g. During the study period, we observed 48 (7.9%) low birth weight (LBW) (< 2500 g) infants and 31 (4.9%) preterm (< 37 weeks of gestation) births (Table 3). Seven (14.6%) of the LBW infants were preterm. Twenty-four [24] of the preterm births had a normal birth weight. Twelve (12 (1.7%)) of the pregnancies resulted in live born twins. From the pregnancies not ending in live birth, 11 (1.6%) terminated without any intervention before 28 weeks of pregnancy. There were three stillbirths, four congenital abnormalities, and two intrauterine foetal deaths.

**Table 3:** Pregnancy outcome characteristics among 704 women in the Adami Tullu district, 2018

Variable	Frequency	Mean (SD)
Birth weight (gm) (*n* = 610)		3214 (525.1)
< 2500	48 (7.9)	
2500–3000	149 (24.4)	
Above 3000	413 (67.7)	
Low birth weight		
Preterm	7 (14.6)	
Term	41 (85.4)	
Gestational age at birth (*n* = 630)		
< 37 weeks	31 (4.9)	
> = 37 weeks	599 (95.1)	
Preterm birth		
Low birth weight	7 (22.6)	
Normal birth weight	24 (77.4)	
Twin pregnancy (*n* = 704)	12 (1.7%)	
Stillbirth (*n* = 658)	3 (0.4%)	

From 48 low birth weight (LBW) new-borns, 12 of them had no ANC. Moreover, out of 31 pregnancies with preterm births, five of them did not have ANC. One of the three pregnancies that resulted in stillbirths had no ANC. Among women who had spontaneous terminations of pregnancy, nine of them had no ANC. Two of the pregnancies who had no sign of life during ultrasound assessment occurred among pregnant women who did not have ANC. In addition, two of the four pregnancies with congenital abnormalities had no ANC (Table 4). The pregnancies with congenital abnormalities were terminated after they were referred to a hospital.

**Table 4:** Adverse pregnancy outcomes and antenatal care utilization in the Adami Tullu district, 2018

Adverse pregnancy outcomes	Antenatal care use	
	Yes	No
Low birth weight		
No	436	126
Yes	36	12
Preterm birth		
No	462	137
Yes	26	5
Intrauterine foetal death	0	2
No	536	166
Yes	0	2
Stillbirth	2	1
No	534	167
Yes	2	1

Table 5 below shows the compliance of antenatal and delivery service utilization with national and WHO guidelines. In our study, we found that the average number of ANC visits was 2.5, which is less than that recommended by the national and WHO guidelines. Only 18 (2.6%) women attended ANC in their first trimester (<=12 weeks), while it is expected to be 100% according to the guidelines. As part of our study, we measured the blood pressure of all pregnant women at each visit and tested them for malaria infection. The guidelines recommend routine measurement of blood pressure for pregnant women at each visit. We did not have information on the actual practices at the health facilities during the study period. Similarly, we did not collect malaria test practices at the health facility level.

**Table 5:** Compliance of ANC and delivery services utilization in comparison with guidelines, Adami Tullu district, 2018

Health service/outcome		Current study (%)	2021 national Ethiopian guidelines (%)	WHO guidelines 2016 (%)
Antenatal care services	No ANC	23.9		
	Number of visits (mean)	2.5	8	8
	BP measurement	100	100	100
	Iron folate supplementation	35	100	100
	TT vaccination^a^	44.7	100	100
	Syphilis test^a^	3.4	100	100
	HIV test^a^	28.7	100	100
Antenatal care done	First trimester	2.60	100	100
	Second trimester	30.90	100	100
	Third trimester	64.10	100	100
Place of antenatal care	Health centre and hospital	79.50	100	100
	Health post	20.50	0	0
Place of delivery	Institutional delivery	47	100	100
	Home delivery	53	0	0

^a^Reported by mothers, *BP* Blood Pressure, *TT* Tetanus Toxoid

According to the study participants’ report, a tetanus toxoid vaccine was given to approximately 45%, an HIV test was done for 29%, and a syphilis test was done for only 3.4% of the women. Iron folate was supplemented for 35% of the women. Even though gestational age is routinely estimated by measuring fundal height using a tape measure, as an ultrasound is not available at the health centres, it was not assessed either in the current study or at the health facilities where routine ANC service is provided. Less than half (47%) of the women delivered in a health facility, in contrast to the 100% expected health institution delivery based on the guidelines. In this study, the number of stillbirths that we found was three (0.4%). It was also shown that assisted delivery (0.6%) and caesarean section delivery (1.3%) were lower than the expected proportion in the national and WHO guidelines [4, 6].

The association between adverse pregnancy outcome and antenatal care service utilization with other explanatory variables is shown in Supplementary Table 3 In this study, adverse pregnancy outcome had no significant association with antenatal care utilization (COR = 1.07, 95% CI 0.62, 1.86, *P* = 0.806).

## Discussion

In this study, the compliance of ANC utilization with national and WHO guidelines was low. It was also found that the current focused ANC utilization did not have an association with adverse pregnancy outcome.

Our results show that the average number of ANC visits in our study was very low (2.5). Only 32% of the pregnant women fulfilled the WHO minimum number of four or more ANC visits. This is lower than the global low and middle income countries (LMIC) proportion of 55% and a report from Myanmar of 59% [34–36]. Those women who initiated ANC during the first trimester were also very low (2.6%) compared to other LMIC, such as Myanmar (47%) [34, 36], and the WHO guideline that expects all pregnant women to start ANC follow-up during the first trimester [2]. More than half (53%) of the women gave birth at home, and 44% of all births were attended by traditional birth attendants.

Most of the pregnant women (76.1%) in our study had attended at least one ANC follow-up, while the number of women with a third and fourth ANC follow-up, which are very important in detecting early signs of complications, was alarmingly low. This is consistent with Ethiopian Demographic and Health Survey 2016, which reported that ANC service utilization was 62%, and the proportion of women who had four ANC visits during their pregnancy was only 32% [10]. However, it is lower than reports from Sub-Saharan Africa, where approximately 80% of pregnant women attended at least one ANC visit, and 52% of pregnant women received the recommended number of four antenatal care visits [37]. The low rate of the fourth ANC follow-up is usually common in studies undertaken in Ethiopia. Women may not be able to attend subsequent follow-ups because of distance, low level of education, and/or poor wealth index [38–40]. This was also documented in studies from other low-income countries [41–44].

The Ethiopian health system delivers primary health care through health centres and their satellite health posts at the community level, and the services are provided free-of-charge. However, in this study, we found that only 21% of the women used ANC services from health posts which were in their vicinity. A previous study from Ethiopia also supports this fact, in which health-care-seeking from health posts was very low, and health centres were preferred over health posts for ANC service by pregnant women [45]. This may be related to the low quality of the services provided and the low trust of women regarding the expertise of the service providers (Health Extension Workers (HEWs)) at the health posts. According to data from a service availability and readiness assessment in 2016, the availability of items for basic facilities, infection prevention, malaria diagnosis, and essential medicines at health posts was low. The general services readiness index at the health post level was 46% [46]. Studies on women’s experiences and satisfaction found that pregnant women’s trust in the skill and competency of HEWs to handle maternal health services was low [47, 48].

In this study, all adverse outcomes were merged, including preterm, intrauterine foetal death and stillbirth, as each of the numbers was small. As a consequence, these findings should be interpreted with caution, as they are not referring to a single adverse outcome. We did not find a significant difference between those mothers who visited health facilities for ANC and those mothers who did not have an ANC follow-up with regard to adverse pregnancy outcome. This is in contrast to other studies [49, 50], which found an association between ANC utilization and pregnancy outcomes. However, other studies have not summarized the adverse outcomes in the same manner as in the present study, and the previous studies have higher numbers of adverse outcomes [51]. One possible explanation for the absence of association between ANC follow-up and pregnancy outcomes could be the low number of ANC follow-ups in the focused or “reduced” ANC model. Only 4.5% of the pregnant women in our study had four ANC visits, while the rest either did not have an ANC follow-up or had less than four ANC follow-ups. Evidence shows that the “standard” ANC model (attending at least eight ANC visits) offers better pregnancy outcomes and a higher likelihood of receiving helpful maternal health interventions when compared to the “focused (reduced) visit” model [52].

Another explanation could be inadequacy in the content and quality of ANC service provided to the pregnant women. A study done on the relationship between ANC and preterm birth reported a significant association between preterm birth and the content and timing of care [53]. Previous literature on the quality of ANC service in public health facilities of Ethiopia showed that the proportion of pregnant women who received adequate ANC content was low [54–56]. They found that a large proportion of the pregnant women missed opportunities to receive blood pressure or weight measurement, and iron/folic acid supplementation. One of the studies also reported poor adherence of the health professionals to the FANC protocol [54].

The proportion of stillbirths in our study (0.4%) was very low compared to previous studies in Ethiopia that reported the proportion of stillbirths to be approximately 4% [55, 57]. The low proportion in our study could be due to under reporting, as there were pregnant women who left the study area for whom the outcome of the pregnancy was not known.

### Strengths and limitations of the study

This study is the first community-based study that followed pregnant women from 24 weeks of gestation up to delivery that was conducted to examine the compliance of antenatal care utilization with national and WHO guidelines, and the association of antenatal health care utilization with adverse pregnancy outcomes in Ethiopia. This contributes to increased knowledge about the implications of antenatal health service utilisation. The second major contribution is the availability of population-based information on prenatal, social, and demographic characteristics collected using maternal interviews and foetal ultrasound during pregnancy.

Our study also has certain limitations. The information collected on ANC service utilization was what was reported by the women. We did not cross check their responses with the medical records. This may introduce misclassification bias. The proportion of women who had one ANC in our study (76.1%) is higher than the national ANC attendance (62%). In addition, the loss to follow up in our study is small (3.2%). The fact that we screened women at home, invited them to enter the study, and advised them to have an ultrasound scan may introduce exposure or intervention bias. That may have contributed to the higher ANC attendance and lower loss to follow up in our study. There are also no reports of diabetes mellitus, HIV, and syphilis that might be potentially contribute for adverse pregnancy outcomes in the study participants. This may affect the generalizability of the findings to a maternal population with a different medical background. Moreover, we did not assess some of the basic indicators of ANC service content and quality, such as nutrition counselling, deworming, and screening for infections that may have an influence on the adverse pregnancy outcomes.

## Conclusions

In conclusion, we found that ANC service utilization is low in the context of national and WHO guidelines. In addition, we did not find any association between the current focused antenatal health care service and adverse pregnancy outcomes.

## Supplementary Information

**Additional file 1: Supplementary Table 1.** Antenatal care utilization by study villages (kebele) in Adami Tullu district, 2018. **Supplementary Table 2.** Antenatal care utilization by sociodemographic characteristics in Adami Tullu district, 2018. **Supplementary Table 3.** Binary logistic regression for ANC utilization with adverse pregnancy outcome (*n* = 704), Adami Tullu district, 2018.

## Data Availability

The datasets used and/or analysed during the current study are available from the corresponding author on reasonable request.

## Abbreviations

ANC: Antenatal care
BP: Blood Pressure
CI: Confidence interval
COR: Crude Odds Ratio
FANC: Focused Antenatal care
gm: Gram
HEP: Health Extension Program
HEW: Health Extension Workers
HIV: Human Immunodeficiency Virus
IFAS: Iron and folic acid supplementation
LBW: Low birth weight
LMIC: Low and Middle Income Countries
SD: Standard Deviation
TEM: Technical errors of measurement
TT: Tetanus Toxoid
WHO: World Health Organization
X^2^: Chi square
