# Supplementary material for: Antenatal care utilization and compliance with national and WHO guidelines in rural Ethiopia: a cohort study
Source: BMC Pregnancy Childbirth. 2022 Nov 17;22:849. doi: 10.1186/s12884-022-05171-3 (PMC9670428; doi:10.1186/s12884-022-05171-3)
Supplement: Supplementary file 1 — Additional file 1: Supplementary Table 1. Antenatal care utilization by study villages (kebele) in Adami Tullu district, 2018. Supplementary Table 2. Antenatal care utilization by sociodemographic characteristics in Adami Tullu district, 2018. Supplementary Table 3. Binary logistic regression for ANC utilization with adverse pregnancy outcome (n = 704), Adami Tullu district, 2018. [file 12884_2022_5171_MOESM1_ESM.docx]

| **Kebele** | **ANC followup** | | **Total** |
| --- | --- | --- | --- |
|  | **No (%)** | **Yes (%)** |  |
| Wolin Bula | 5 (19.2) | 21(80.8) | 26 |
| Negalegn | 3 (12.5) | 21(87.5) | 24 |
| Ilka Chelemo | 13 (7.8) | 60 (82.2) | 73 |
| Edo Gojola | 14 (28.0) | 36 (72.0) | 50 |
| Abine Geremama | 15 (23.8) | 48 (76.2) | 63 |
| Qamo Garbi | 2 (12.5) | 14 (87.5) | 16 |
| Garbi Widena Boramo | 4 (28.6) | 10 (71.4) | 14 |
| Halaku | 3 (14.3) | 18 (85.7) | 21 |
| Anano Shisho | 15 (18.5) | 66 (81.5) | 81 |
| Golba Aluto | 11 (17.7) | 51 (82.3) | 62 |
| Dodicha | 13 (24.1) | 41 (75.9) | 54 |
| Boccessa | 24 (34.8) | 45 (65.2) | 69 |
| Abayi Deneba | 46 (30.5) | 105 69.5) | 151 |
| Total | 168 (23.9) | 536 (76.1) | 704 |

Supplementary Table 1. Antenatal care utilization by study villages (kebele) in Adami Tullu district, 2018

Supplementary Table 2. Antenatal care utilization by sociodemographic characteristics in Adami Tullu district, 2018

| Variable | **Yes (n (%))** | **No (n (%))** | **X^2^ (P-value)** |
| --- | --- | --- | --- |
| Age  <20  20-24  25-34  35 and above | 76 (71.0)  184 (77.3)  243 (77.9)  33 (70.2) | 31 (29.0)  54 (22.7)  69 (22.1)  14 (29.8) | 3.150 (0.369) |
| Parity  Nullpara  Primipara  Mutipara | 101 (76.5)  102 (72.3)  333 (77.3) | 31 (23.5)  39 (27.7)  98 (22.7) | 1.429 (0.489) |
| Educational status  Illiterate  Read and write  Primary  Secondary | 238 (75.1)  32 (74.4)  223 (78.5)  43 (71.7) | 79 (24.9)  11 (25.6)  61 (21.5)  17 (28.3) | 1.814 (0.612) |
| Occupation  Housewife  Farmer  Housemaid  Others | 411 (76.1)  95 (79.2)  10 (58.8)  125 (76.2) | 129 (23.9)  25 (20.8)  7 (41.2)  39 (23.8) | 3.474 (0.324) |
| Wealth status  Poor  Middle  Rich | 170(32.0)  178 (33.5)  183 (34.5) | 60 (35.9)  59 (35.3)  48 (28.7) | 1.969 (0.374) |
| Intended place of delivery  Home  Health institution | 110 (72.8)  382 (79.1) | 41 (27.2)  101 (20.9 | 2.578 (0.108) |
| Place of delivery  Home  Health institution | 261 (77.4)  231 (77.8) | 76 (22.6)  66 (22.2) | 0.010 (0.921) |

Supplementary Table 3. Binary logistic regression for ANC utilization with adverse pregnancy outcome (n=704), Adami Tullu district, 2018

|  |  | **Adverse pregnancy outcome** | |  |  |
| --- | --- | --- | --- | --- | --- |
| **Characteristics** | | **Yes** | **No** | **Crude OR** | ***P*-value** |
| Age |  |  |  |  |  |
|  | < 24 yrs | 30 | 272 | 1 |  |
|  | >= 24 yrs | 46 | 356 | 1.17 (0.72, 1.91) | 0.523 |
| Educational status | |  |  |  |  |
|  | No formal | 33 | 327 | 0.71 (0.44, 1.14) | 0.156 |
|  | Formal | 43 | 301 | 1 |  |
| Occupation |  |  |  |  |  |
|  | Housewife | 60 | 480 | 1.16 (0.65, 2.07) | 0.625 |
|  | Others | 16 | 148 | 1 |  |
| Gravida |  |  |  |  |  |
|  | Primi gravida | 15 | 98 | 1 |  |
|  | Multigravida | 61 | 530 | 0.75 (0.41, 1.38) | 0.335 |
| Parity |  |  |  |  |  |
|  | Null para | 18 | 114 | 1 |  |
|  | Multipara | 58 | 514 | 1.39 (0.79, 2.46) | 0.245 |
| DBP |  |  |  |  |  |
|  | 80-89 | 6 | 35 | 0.69 (0.28, 1.69) | 0.417 |
|  | <80 | 70 | 593 | 1 |  |
| Maternal haemoglobin |  |  |  |  |  |
|  | <=10.99 | 16 | 119 | 1 |  |
|  | >=11 | 60 | 509 | 1.14 (0.64, 2.05) | 0.660 |
| Maternal height |  |  |  |  |  |
|  | <150 | 12 | 61 | 0.57 (0.29, 1.12) | 0.102 |
|  | >=150 | 63 | 561 | 1 |  |
| Wealth status |  |  |  |  |  |
|  | Poor | 23 | 207 | 0.84 (0.47,1 .51) | 0.560 |
|  | Middle | 25 | 212 | 0.89 (0.50, 1.59) | 0.690 |
|  | Rich | 27 | 204 | 1 |  |
| ANC utilization |  |  |  |  |  |
|  | Yes | 57 | 479 | 1 |  |
|  | No | 19 | 149 | 1.07 (0.62,1.86) | 0.806 |
